# 375. High Laboratory-confirmed SARS-CoV-2 Attack Rate in Lima Health Care Personnel During August 2020-March 2021 Suggests Role for Improved Infection Control

**DOI:** 10.1093/ofid/ofab466.576

**Published:** 2021-12-04

**Authors:** Matthew Westercamp, Giselle Soto, Rachel Smith, Eduardo Azziz-Baumgartner, Susan Bollinger, Roger Castillo, Alejandro Llanos Cuentas, Max Grogl, Natalie Olson, Mike Prouty, Eduardo Matos, Candice Romero, Marita Silva, Fernanda C Lessa, Fernanda C Lessa, Carmen S Arriola

**Affiliations:** 1 Centers for Disease Control and Prevention, Atlanta, Georgia; 2 U.S. Naval Medical Research Unit No. 6, Lima, Peru; 3 U.S. Centers for Disease Control and Prevention, Atlanta, Georgia; 4 Hospital Nacional Cayetano Heredia, Lima, Peru; 5 U.S. Centers for Disease Control and Prevention – Influenza Division, Atlanta, Georgia; 6 Hospital Nacional Arzobispo Loayza, Lima, Peru; 7 Centers for Disease Control and Prevention, Atlanta, GA

## Abstract

**Background:**

Peru has one of the highest per capita SARS-CoV-2 death rates in Latin America. Healthcare workers (HCW) are a critical workforce during the COVID-19 pandemic but are themselves often at increased risk of infection. We evaluated SARS-CoV-2 attack rate and risk factors among frontline HCWs.

**Methods:**

We performed a prospective cohort study of HCW serving two acute care hospitals in Lima, Peru from Aug 2020 to Mar 2021. Participants had baseline SARS-CoV-2 serology using the CDC ELISA, active symptom monitoring, and weekly respiratory specimen collection with COVID-19 exposure/risk assessment for 16-weeks regardless of symptoms. Respiratory specimens were tested by real-time reverse transcriptase PCR (rRT-PCR).

**Results:**

Of 783 eligible, 667 (85%) HCW were enrolled (33% nurse assistants, 29% non-clinical staff, 26% nurses, 7% physicians, and 6% other). At baseline and prior to COVID-19 vaccine introduction, 214 (32.1%; 214/667) were reactive for SARS-CoV-2 antibodies. In total, 72 (10.8%; 72/667) HCWs were found to be rRT-PCR positive during weekly follow-up. Of the rRT-PCR positive HCWs, 37.5% (27/72) did not report symptoms within 1-week of specimen collection. During follow up, HCW without detectable SARS-CoV-2 antibodies at baseline were significantly more likely to be rRT-PCR positive (65/453, 14.3%) compared to those with SARS-CoV-2 antibodies at baseline (4/214, 1.9%) (p-value: < 0.001). Three HCW were both serologically reactive and rRT-PCR positive at baseline. Looking only at HCW without SARS-CoV-2 antibodies, nurse assistants (rRT-PCR positive: 18.6%; 27/141) and non-clinical healthcare workers (16.5%; 21/127) were at greater risk of infection compared to nurses (8.5%; 10/118), physicians (7.9%; 3/38), and other staff (10.3%; 4/29) (RR 1.95;95%CI 1.2,3.3; p-value: 0.01).

**Conclusion:**

Baseline SARS-CoV-2 prevalence and 16-week cumulative incidence were substantial in this pre-vaccination Peruvian HCW cohort. Almost 40% of new infections occurred in HCW without complaint of symptoms illustrating a limitation of symptom-based HCW screening for COVID-19 prevention. Nurse assistants and non-clinical healthcare workers were at greater risk of infection indicating a role for focused infection prevention and risk reduction strategies for some groups of HCW.

**Disclosures:**

**Fernanda C. Lessa, MD, MPH**, Nothing to disclose

